# Atherosclerotic disease is the predominant aetiology of acute coronary syndrome in young adults

**DOI:** 10.5830/CVJA-2017-035

**Published:** 2018

**Authors:** Pillay AK, Naidoo DP

**Affiliations:** Department of Internal Medicine, University of KwaZulu- Natal, Durban, South Africa; Department of Cardiology, University of KwaZulu-Natal, Durban, South Africa

**Keywords:** coronary artery disease, young adults, risk factors, metabolic syndrome

## Abstract

**Objectives:**

Few studies have evaluated young adults in their third and fourth decades with coronary artery disease (CAD). This study evaluated the clinical and angiographic profile of young adults (< 35 years) with CAD.

**Methods:**

A 10-year (2003–2012) retrospective chart reviewwas performed on patients less than 35 years diagnosed withCAD at Inkosi Albert Luthuli Central Hospital, Durban.

**Results:**

Of the 100 patients who met the study criteria, the majority were male (90%), of Indian ethnicity (79%), and presented with acute coronary syndrome (93%). Smoking (82%), dyslipidaemia (79%) and dysglycaemia (75%) were the most prevalent risk factors. Almost half of the subjects (48%) met criteria for the metabolic syndrome. Angiographic findings revealed multi-vessel (42%), single-vessel (36%) and non-occlusive disease (20%); only two subjects had normal epicardial vessels. Disease severity was influenced by dyslipidaemia (p = 0.002) and positive family history (p = 0.002). Non-coronary aetiologies were identified in 19% of subjects.

**Conclusions:**

Atherosclerotic disease associated with risk-factor clustering was highly prevalent in young adults with CAD.

Coronary artery disease (CAD) is considered premature when it appears in adults under the age of 55 years in males and 65 years in females. Premature coronary artery disease (PCAD) is an emerging problem, frequently presenting as premature myocardial infarction (MI) in recent years.^1^,^2^ A recent review of young adults (< 40–45 years)^3^ with CAD described two forms of the disease, one characterised by limited (single) vessel disease with a favourable outcome, and the other by extensive multi-vessel involvement with a more rapid progression of atherosclerosis.

Coronary atherosclerosis, beginning as a fatty streak and raised atheromatous plaque, has been noted to begin early in adolescence,^4^ the majority of patients remaining asymptomatic until later in life.^3^ This silent process makes the estimation of disease prevalence a particular challenge in young adults.^3^ Although a prevalence of four to 10% has been reported among individuals with myocardial infarction under age 40–45 years,^5^,^6^ autopsy studies have found advanced coronary atheroma in up to 20% of men and 8% of women between 30 and 34 years of age.^7^ In a local study by Ranjith et al., 20% (n = 491) of 2 290 patients presenting with MI, between 1996 and 2002 were under the age of 45 years.^8^ This increased cardiovascular risk in youth has been noted to be particularly high among the South African Indian community in whom risk-factor clustering has been described.^8^-^10^

In contrast to older subjects, major cardiovascular risk factors such as hypertension and diabetes mellitus are less commonly observed among young adults with PCAD.^11^ Subtle forms of dysglycaemia such as insulin resistance and impaired glucose tolerance have been found to be more common than diabetes in this age group and add to the risk of PCAD.^12^ Risk-factor clustering in the form of the metabolic syndrome has also been reported to be common among young patients.^11^ Additional, ‘non-conventional’ risk factors may also be more commonly found among younger subjects. These include psychosocial factors such as stress^12^-^14^ and anger,^15^ the use of recreational drugs such as cocaine^16^ and marijuana,^17^ connective tissue disease^18^,^19^ and HIV infection.^20^,^21^

Earlier studies have documented at least one major risk factor in over 90% of young subjects with CAD;^22^,^2^ more recently the INTERHEART study^24^ identified major risk factors in subjects with CAD worldwide among young subjects. A corresponding increase in mortality rate has also been associated with an increasing number of risk factors.^25^ Little is known about the underlying aetiology and angiographic profile of young subjects presenting with CAD. In this study we analysed the clinical and angiographic profile of young adults (< 35 years) presenting to the Cardiology Unit at Inkosi Albert Luthuli Central Hospital over a 10-year period.

## Methods

A retrospective chart review was conducted on young patients (<35 years) with CAD referred to the Cardiology Department atInkosi Albert Luthuli Central Hospital (IALCH) over a 10-yearperiod between 2003 and 2012. All patients were referred with adiagnosis of acute coronary syndrome (ACS) or stable angina.The diagnosis of ACS was made according to criteria outlinedby Braunwald et al. and encompasses unstable angina, non-STsegmentelevation (non-Q wave) MI (NSTEMI) and ST-segmentelevation (Q wave) MI (STEMI).^26^

The ethics committee of the Faculty of Health Sciences, Nelson R Mandela School of Medicine, University of KwaZulu- Natal granted approval for the study (BE324/13).

As per unit policy, all young subjects diagnosed with ACS/CAD undergo coronary angiography. Patients referred forcoronary angiography for reasons other than assessment of CAD(such as chest trauma or prior to elective valve replacement) werenot included. Patients were identified using the Speedminersoftware program, which is a Data Warehouse Managementsoftware package, used by the hospital to manage, process andcategorise the data collected on its database. All patient chartswere accessed via the software program and data were extractedon demographics, clinical and biochemical parameters, as well asinvestigations including SestaMIBI and coronary angiography.

Clinical and biochemical parameters were assessed to determine the risk-factor profile as well as factors that could influence the clinical outcome of patients. In addition to the metabolic syndrome criteria (see below), other parameters included in the analysis were: body mass index, total cholesterol > 4.5 mmol/l, low-density lipoprotein cholesterol (LDL-C) > 1.8 mmol/l, glycated haemoglobin (HbA1c) > 6.5%, haemoglobin < 13 g/dl for males, < 12 g/dl for females, microalbuminuria 30–300 mg/l and proteinuria > 300 mg/l.

The International Diabetes Federation consensus criteria (harmonised definition) (2006) were used to detect subjects with the metabolic syndrome (MetS)^27^ when they had at least three of the following factors: central obesity [waist > 94 cm in males (90 cm in Indians) and > 80 cm in females], triglycerides > 1.7 mmol/l, high-density lipoprotein cholesterol (HDL-C) < 1.03 mmol/l in males or < 1.29 mmol/l in females, blood pressure > 130/85 mmHg (or previously diagnosed hypertension) and fasting plasma glucose > 5.6 mmol/l (or previously diagnosed type 2 diabetes mellitus).

Coronary stenosis of ≥ 50% in any of the major coronary arteries was designated occlusive CAD, and stenosis of < 50% non-occlusive coronary disease (NOD). For scoring the severity of CAD, luminal stenosis of 50% of the proximal coronary artery was given a score of 1, 50–74% of 2, 75–99% was scored as 3 and total occlusion was scored as 4.28

## Statistical analysis

Statistical Package for the Social Sciences (SPSS version 23.0) was used for data analysis. A 95% confidence interval (CI) was estimated and a global significance level of α = 5% was chosen. Simple descriptive analysis was used to identify clinical characteristics and present results as frequencies, means and percentages.

The chi-squared test and Mann–Whitney U-test were used for categorical variables to determine the relationship between discontinuous variables or continuous variables in assessing the significance of risk factors between subjects with and without angiographic CAD. Binary logistic regression analysis was used to analyse confounding factors when assessing the independent relationships between risk factors and the outcome variable (CAD). A two-way ANOVA analysis was used to assess the effect of clinical criteria and other risk factors on the presence or absence of the metabolic syndrome and the likelihood of CAD.

## Results

During the 10-year study period (January 2003 to December 2012), 7 575 subjects with CAD underwent coronary angiography. Among this group, 100 subjects were 35 years or younger, constituting 1.3% of all subjects with coronary disease referred for coronary angiography. These subjects (90 males, 10 females) had a mean age of 31.9 years (median 27.5 years) and 23 were under 30 years of age. The ethnic distribution showed a predominance of Indian subjects (79%) (Table 1).

**Table 1:** Demographic profile of the patients

*Characteristics*	*n = 100*
Median age (years)	33
Ethnicity	
Indian	79
White	11
Black	7
Coloured	3
Presentation
STEMI	66
NSTEMI	13
Chronic stable angina	6
Unstable angina	14
Symptomatic bradycardia	1
Risk profile
Smoking	82
Hypertension	28
Diabetes	26
Dyslipidaemia	80
Obesity	30
Family history	74
Illicit drug use	8
Retroviral disease	2
Systemic lupus erythematosus	1

NSTEMI: non-ST-segment elevation MI; STEMI: ST-segment elevation MI.

The majority of subjects (n = 93) were referred from their base hospital following a diagnosis of acute coronary syndrome. Most (82%) presented with ‘typical’ acute ischaemic chest pain, while atypical chest pain symptoms (sharp, stabbing pain; symptoms suggestive of dyspepsia or heartburn) were reported in 18 cases (18%).

Sixty-six patients (66%) had STEMI, 45 of whom received thrombolytic therapy on admission at the base hospital prior to referral. The reason most often cited for failure to administer thrombolysis in the remaining 21 patients was late presentation (> 24 hours since onset of chest pain). A further 13 patients were referred with NSTEMI, and 14 patients presented with unstable angina. Six subjects presented with chronic stable angina and were referred following positive exercise stress tests. One patient presented with symptomatic bradycardia (complete heart block) (Table 1).

Varying combinations of cardiovascular risk factors were present (Table 1) in all but one subject, a 32-year-old black male who presented with chronic stable angina, no cardiovascular risk factors and he had single-vessel disease at angiography.

The two most common risk factors identified were smoking (82%) and dyslipidaemia (79%) (Table 1). The dyslipidaemia comprised hypercholesterolaemia (67.4%), hypertriglyceridaemia (63.7%) and low HDL-C (56.5%). In the 87 patients in whom LDL-C could be calculated by the Friedewald formula, 78 (90%) were found to have levels greater than 1.8 mmol/l (Table 2). The atherogenic combination of raised triglycerides (TG) and low HDL-C was found in 30 subjects.

**Table 2:** Biochemical profile of subjects

*Biochemical profile*	*Number*	*Number Percentage*
Lipids
TC > 4.5 mmol/l	62/92	67
TG > 1.7 mmol/l	58/91	63
HDL-C < 1 mmol/l (M), 1.2 mmol/l (F)	52/92	56
LDL-C > 1.8 mmol/l	78/87	90
Glucose
Fasting glucose > 5.6 mmol/l	49/99	49.5
HbA1c > 6.5%	25/97	26
Hb < 13 g/dl (M), 12 g/dl (F)	10/100	10
Microalbuminuria	24/32	75

TC: total cholesterol; TG: triglycerides; HDL-C: high-density lipoprotein cholesterol; LDL-C: low-density lipoprotein cholesterol; HbA1c glycated haemoglobin; Hb: haemoglobin.

There were 41 patients who were classified as overweight (BMI > 25 kg/m^2^, > 23 kg/m^2^ in Indians) and 30 subjects (30%) were classified as obese (BMI > 30 kg/m^2^). Increased waist circumference was found in 44 patients (42 males and two females). On applying the ethnic-specific harmonised criteria, 48 subjects (48%) were found to have the MetS. The most prevalent criteria in these subjects were waist circumference, impaired fasting glucose level and hypertriglyceridaemia (Table 3). Among the subjects without the MetS (n = 52), dyslipidaemia was present in over 40% and there was a high prevalence of overweight or obesity (65%), a positive family history for CAD (73%) and smoking (83%).

**Table 3:** Characteristics of subjects with the metabolic syndrome

*Characteristics*	*MetS (n = 48)*	*No MetS (n = 52)*	*Total (n = 100)*
MetS criteria
WC > 94 (90)/80	46	14	60
BP > 130/80	14	9	23
FPG > 5.6	41	12	53
TG > 1.7	39	23	62
HDL < 1.0/1.2	30	21	51
Other factors
BMI > 25 (23)	47	34	81
Family history	38	38	76
Smoking	40	43	83
Gender
Male	43	47	90
Female	5	5	10
Race
Indian	41	38	79
White	3	8	11
Black	2	5	7
Coloured	2	1	3

WC: waist circumference; BP: blood pressure; FPG: fasting plasma glucose; TG: triglycerides; HDL: high-density lipoprotein; BMI: body mass index.

Hypertension and diabetes mellitus were present in 28 and 26% of subjects, respectively. There were 20 known (type 1 diabetes mellitus, n = 1) and six newly diagnosed subjects with type 2 diabetes mellitus. On biochemistry, impaired fasting glucose (> 5.6 mmol/l) was present in a further 49 subjects, yielding a 75% prevalence of dysglycaemia in these subjects (Table 2). The average HbA1c level among subjects with known diabetes was 10.0%, indicating a poor level of glycaemic control in these subjects.

Seventy-four (74%) subjects had a family history of CAD. Of these, a history of premature CAD in the immediate family [a first-degree relative under age 55 (males) or 65 (females)] was documented in 44 subjects.

Non-conventional risk factors were found in 19 subjects. Of these, illicit drug use (n = 8), retroviral disease (n = 2) and alcohol excess (n = 2) were the main factors (Table 4). Of interest, there was one subject with systemic lupus erythematosus and one with hypothyroidism. Two patients had co-existing rheumatic valvular heart disease, but they did not have infective endocarditis or atrial fibrillation as predisposing factors for coronary embolism.

**Table 4:** Non-conventional risk factors

*Non-coronary risk factors*	*Number*
Drug use	8
Alcoholism	2
Valvular heart disease	2
HIV	2
SLE	1
Oral contraceptives	1
Hypothyroidism	1
Post-operative	1
Thrombophilia (suspected)	1

SLE: systemic lupus erythematosus

Four of the five patients who were ≤ 25 years at the time of presentation, had a positive family history of premature CAD and dyslipidaemia (Table 5). The fifth subject was a 25-year-old black male patient with a history of smoking and illicit drug use who had normal epicardial coronary arteries at angiography. It was suspected that the aetiology in this patient was coronary spasm related to drug use.

**Table 5:** Risk-factor comparison across age groups

	*Age range, years (n)*		
*Risk factors*	*20–25 (5)*	*26–30 (18)*	*31–35 (77)*
Smoking, n (%)	1 (20)	12 (67)	69 (90)
Hypertension, n (%)	1 (20)	7 (39)	20 (26)
Diabetes, n (%)	1 (20)	3 (17)	22 (29)
Dyslipidaemia, n (%)	4 (80)	16 (89)	60 (78)
Obesity, n (%)	0	8 (44)	22 (29)
Family history, n (%)	4 (80)	12 (67)	58 (75)
Drugs, n (%)	1 (20)	2 (11)	7 (9)

The youngest subject was a 20-year-old Indian male whopresented with an anterior STEMI and was subsequently foundto have non-occlusive disease on angiography with a kinkeddistal left anterior descending (LAD) artery. There was noevidence of hypertrophic obstructive cardiomyopathy (HOCM)in this case. One Indian patient, aged 25 years, had a long historyof uncontrolled type 1 diabetes mellitus and hypertension. Theremaining two subjects (22-year-old coloured and 24-year-oldIndian males) had three-vessel disease at angiography.

On clinical examination, signs of heart failure (elevated jugulovenous pressure, lower-limb oedema, pulmonary crepitations) were identified in 5% of subjects on presentation. Arrhythmias were identified in eight subjects, four with tachyarrhythmias [supraventricular tachycardia (n = 1), ventricular tachycardia (n = 1), ventricular fibrillation (n = 2)] and four with bradyarrhythmias [first-degree (n = 1), seconddegree (n = 2) complete heart block (n = 1)].

The admission electrocardiogram (ECG) revealed that the majority of patients (65%) had evidence of anterior ischaemia or infarction; further classified as anterior (n = 9), anterolateral (n = 32) and anteroseptal (n = 24). Thirty per cent presented with inferior (n = 15), inferolateral (n = 13) or inferoposterior (n = 2) involvement. Fully evolved Q waves were identified in 63% of subjects, likely indicative of late presentation as none had a previous history of coronary events.

Echocardiography revealed regional wall motion abnormalities in 83% of subjects; the ejection fraction (EF) was < 50% in 42%, with evidence of left ventricular thrombus in nine patients. Reversible ischaemia was identified in 19/29 (65%) subjects who underwent technetium (99mTC) SestaMIBI scanning.

Coronary angiography revealed occlusive CAD (> 50% stenosis) in 78 subjects, while 20% had non-occlusive disease and the remaining two subjects had normal epicardial vessels. Single-vessel disease was present in 36 subjects, with the LAD artery being the most commonly involved vessel (n = 33, 92%). Multi-vessel disease was found in 42 subjects; of whom 27 had two-vessel disease (19 with LAD involvement) and 15 had threevessel disease.

Among the 26 subjects with diabetes mellitus, angiography revealed most (n = 12) had multi-vessel disease, followed by singlevessel (n = 7) and non-occlusive disease (n = 6). Atherosclerotic coronary disease was present in five of the eight subjects with a history of illicit drug use. There were two subjects who had normal epicardial vessels at coronary angiography: one was the 25-year-old black male who had a history of illicit drug use including cocaine, and the second was a 34-year-old HIV-positive black male. Both subjects presented with anterior STEMI and received thrombolysis at their base hospitals prior to referral.

To determine the association of various risk factors on the severity of stenosis or number of vessels affected, we conducted a Mann–Whitney U-test with stenosis severity or number of vessels as the dependent variable and major risk factors as the independent variable. Dyslipidaemia was associated with severity of stenosis (p = 0.002) as well as the number of vessels involved (p = 0.039). Low HDL-C was particularly associated with disease severity (p = 0.004). A positive family history was also found to be associated with both severity of stenosis (p = 0.002) and number of vessels involved (p = 0.001). Hypertension (p = 0.36), diabetes (p = 0.88), the MetS (p = 0.80) and smoking (p = 0.70) were not associated with disease severity. An association with severity and increased waist circumference (p = 0.08) and generalised obesity (p = 0.08) was shown but this was not significant.

To determine the relationship between risk factors and occlusive CAD, a chi-squared test was conducted with dyslipidaemia, obesity, smoking, family history, hypertension, diabetes or the MetS as independent factors and occlusive CAD as the dependent factor. On bivariate analysis, a strong association between dyslipidaemia and occlusive CAD was observed (χ2 = 11.717, p = 0.001, RR = 5.52) while major risk factors such as hypertension (p = 0.30), diabetes (p = 0.59) smoking (p = 0.14), family history (p = 0.16) and the MetS (p = 0.93) were not associated with occlusive CAD.

To determine the effect of the MetS in combination with other risk factors on the severity of CAD, a two-way ANOVA analysis was conducted with the coronary artery score as the dependent variable and the MetS as the grouping variable, along with various risk factors. Generalised obesity (BMI > 30 kg/m^2^) in combination with the MetS appeared to influence severity of stenosis (p = 0.004); however, a strong association was not demonstrated with smoking (p = 0.85) or family history of CAD (p = 0.591). When assessed independently of the MetS, the combination of raised triglycerides and low HDL-C influenced severity of stenosis (p = 0.05) but not number of vessels involved (p = 0.33).

To further assess the association of various risk factors with significant CAD, a binomial regression analysis was conducted with significant CAD as the dependent variable and gender, ethnicity, cardiovascular risk factors and the presence or absence of the MetS as covariates. For ethnicity, three dummy variables were created and compared with Indians as the baseline; similarly with regard to age, an age range of 20−24 years was taken as baseline for comparison, and 25−30 and 31−35 years were assigned dummy variables. Only dyslipidaemia showed a significant association with occlusive CAD (p = 0.008, OR: 0.21, 95% CI: 0.670–0.672).

## Discussion

In this study, young adults comprised 1.3% of subjects with CAD referred for coronary angiography, and the majority presented with acute coronary syndrome. While often regarded as a disease of advancing age, atherosclerotic changes in the coronary vessels have been documented early in adolescence,^29^ with changes in lifestyle and dietary habits30-32 contributing to CAD becoming clinically manifest early in the third decade of life,^8^ particularly among certain ethnic groups such as the Indian population.^8^-^10^

The observation that CAD prevalence differs significantly among ethnic groups is in agreement with earlier studies that have shown a 50% higher risk of CAD among expatriate Indians compared to other ethnic groups such as Hispanics and blacks,^33^ even after adjusting for lifestyle factors.^34^ The majority of subjects in our study were of Indian origin (79%), of whom 53 (81%) were diagnosed with occlusive CAD. The data are also in agreement with the CADI study, which estimated a higher risk of CAD among Indians.^35^

Our findings suggest that young patients are less likely to present with symptoms of stable angina,^36^ their first manifestation of CAD being most often an ACS, which untreated or unrecognised, progressed rapidly to MI, STEMI in particular.^37^,^38^ Up to two-thirds of young subjects deny a history of chest pain prior to MI;39 when present, angina symptoms have been reported to occur most often in the week preceding the event.^37^ A study of 200 subjects under 45 years of age with angiographic CAD found a lower incidence of stable angina (24%) and a higher incidence of ACS (76%) compared to subjects over 60 years, with a higher likelihood of complex lesions on angiogram.^38^

Similar to previous studies in young subjects,^40^-^43^ smoking was highly prevalent in our sample, and conferred a greater risk (OR 2.9) among Indian and white subjects. Our findings also confirm a male preponderance in young subjects with CAD,^37^,^44^,^45^ which has been attributed the higher prevalence of smoking among young men and to non-modifiable factors such as the protective effect of oestrogen in women.^25^ The 82% prevalence of smoking in our study is in keeping with registry data of patients with STEMI undergoing percutaneous coronary intervention (PCI) where smoking rates were highest among the age range of 18–34 years (78%) compared both to older age groups and the general population of similar age (23%).^46^ Since other cardiovascular risk factors were also highly prevalent in our study, it is likely that smoking acted in concert with these factors to result in CAD.

Our study supports the finding that clustering of major cardiovascular risk factors predominates in young patients with CAD.^47^,^48^ In addition to smoking, dyslipidaemia (80%) and a positive family history of CAD (74%) were the most frequent risk factors identified.

Analysis of the lipid profile showed that elevated LDL-C was present in 90% of the 87 subjects in whom it could be calculated. The atherogenic lipid profile of raised triglycerides and low HDL-C levels was present in 30% of the sample, and 30% were classified as obese. Major risk factors including hypertension and diabetes mellitus (28 and 26%, respectively) were frequently present in this young cohort of subjects with PCAD, compared to previous studies of older subjects in this population.^49^ Of importance, we have noticed the emergence of illicit drug use (cannabis, heroin, cocaine and the local street drug ‘sugars’ containing a mixture including cocaine residue) as a contributory risk factor in 8% of subjects.

The third most prevalent risk factor among our subjects was a positive family history of CAD (74%), which influenced both the extent and severity of CAD (p = 0.045 and p = 0.002, respectively). It is well documented that young subjects with CAD more often have a positive family history than middle-aged or elderly patients,^50^-^53^ with contributions to this increased risk from both genetic and environmental factors. In a cohort similar to ours, Ranjith et al. found a family history of premature CAD in 54% of South African Indians with MI.^11^ Parental CAD was a strong predictor of MI in offspring in the INTERHEART study, suggesting that in addition to possible genetic factors, similar environmental exposure contributed to type 2 diabetes mellitus, hypertension and obesity and the increased cardiovascular risk.^46^,^51^

Our findings of high prevalence of visceral obesity, high triglyceride and low HDL-C levels, together with elevated LDL-C and dysglycaemia, suggest environmental factors as a major contributor to the emergence of PCAD in young adults in their third decade of life. The combination of subtle abnormalities of glucose metabolism^12^ with clustering of other risk factors that comprise the MetS has been recognised as a significant predictor of CAD.^54^

The prevalence of the MetS has been documented to differ significantly among ethnic groups^54^ and between age groups, rising from less than 10% in the 20–29-year age group to between 38 and 67% in the 60–69-year age group.^55^ Almost half the subjects in our study (48%) met the modified IDF criteria^27^ for the MetS.

In a previous study, Ranjith et al. assessed the prevalence of the MetS among young (< 45 years) South African Indian subjects with MI using the NCEP ATP III and IDF criteria, and found between 57 and 60% of subjects met the criteria respectively.^56^ This study suggested that use of the modified IDF ethnic-specific waist circumference cut-off points as the determinant of abdominal obesity was more useful to accurately identify patients in this population group. Waist circumference was the main driver (44/48) for the MetS in our study, reflecting visceral adipose tissue as a major contributor to the increased risk of hyperinsulinaemia, insulin resistance, diabetes and dyslipidaemia in this population.^55^

Our finding of higher coronary artery severity (CAS) scores in association with a positive family history of PCAD and dyslipidaemia (low HDL-C in particular) is in agreement with earlier observations.^57^ A strong association has been shown between dyslipidaemia and the presence of occlusive CAD (p = 0.004), as well as severity of disease (p = 0.002). Although type 2 diabetes mellitus is known to be a strong predictor of CAD, particularly among groups usually considered ‘low risk’, such as young patients, women and non-smokers,^58^ it did not influence the extent (p = 0.56) or severity of disease (p = 0.88) in our study, probably due to the shorter duration of diabetes in our cohort of younger subjects, less than 35 years. In contrast to previous studies that have shown the MetS to be associated with extensive (three-vessel) disease,^56^ neither smoking nor the presence of the MetS contributed significantly to the severity of CAD.

Multi-vessel involvement was a characteristic angiographic pattern in our study, with only a third of subjects having single-vessel disease, in contrast to the findings of the CASS study, which found a higher frequency of non-occlusive and single-vessel disease in young subjects,^37^ The LAD was the most frequently involved coronary vessel in both groups, as noted in a previous study.^59^

## Limitations

Our study was limited to a specific geographical area and, more specifically, to a single tertiary referral centre but cases were referred from throughout the province of KwaZulu-Natal. We found a much higher prevalence of PCAD among Indians, and although this ethnic group does not represent a majority in the province concerned, the community is largely concentrated in the Durban area. Because of a small sample size, we could not undertake age and gender matching across race groups, limiting comparisons on gender and ethnic differences in risk factors. Fewer ‘conventional’ cardiovascular risk factors, common in the older population, were found to have a statistically significant relationship with PCAD in very young patients. Among the factors that may have contributed to this indeterminate result include the age range studied and the sample size, which was not gender matched.

## Conclusion

This study shows that over two-thirds of young subjects referred to a tertiary centre for coronary angiography due to acute ischaemic chest pain symptoms had atherosclerotic multi-vessel disease. The predominance of major modifiable risk factors suggests high environmental exposure in young adults and calls for early lifestyle changes, beginning at school-going age.

## References

[1] Mohammad AM, Jehangeer HI, Shaikhow SK (2015). Prevalence and risk factors of premature coronary artery disease in patients undergoing coronary angiography in Kurdistan, Iraq. BMC Cardiovasc Disord.

[2] Ranjith N, Pegoraro R, Naidoo D (2004). Demographic data and outcome of acute coronary syndrome in the South African Asian Indian population. Cardiovasc J S Afr.

[3] Klein LW, Nathan S (2003). Coronary artery disease in young adults. J Am Coll Cardiol.

[4] Tuzcu EM, Kapadia SR, Tutar E (2001). High prevalence of coronary atherosclerosis in asymptomatic teenagers and young adults evidence from intravascular ultrasound. Circulation.

[5] Doughty M, Mehta R, Bruckman D (2002). Acute myocardial infarction in the young – The University of Michigan experience. Am Heart J.

[6] Savji N, Rockman CB, Skolnick AH (2013). Association between advanced age and vascular disease in different arterial territories: a population database of over 3.6 million subjects. J Am Coll Cardiol.

[7] McGill HC, McMahan CA, Zieske AW (2000). Association of coronary heart disease risk factors with microscopic qualities of coronary atherosclerosis in youth. Circulation.

[8] Ranjith N, Pegoraro R, Naidoo D (2004). Demographic data and outcome of acute coronary syndrome in the South African Asian Indian population. Cardiovasc J S Afr.

[9] Ranjith N, Verho N, Verho Ms, Winkelmann B (2002). Acute myocardial infarction in a young South African Indian-based population: patient characteristics on admission and gender-specific risk factor prevalence. Curr Med Res Opin.

[10] Ranjith N, Pegoraro RJ, Zaahl MG (2012). Risk factors associated with acute coronary syndromes in South African Asian Indian patients (The AIR Study). J Clin Exper Cardiol.

[11] Cole JH, Miller JI, Sperling LS, Weintraub WS (2003). Long-term follow-up of coronary artery disease presenting in young adults. J Am Coll Cardiol.

[12] Malmberg K, Båvenholm P (1994). Clinical and biochemical factors associated with prognosis after myocardial infarction at a young age. J Am Coll Cardiol.

[13] Holmes SD, Krantz DS, Rogers H, Gottdiener J, Contrada RJ (2006). Mental stress and coronary artery disease: a multidisciplinary guide. Prog Cardiovasc Dis.

[14] Kivimäki M, Virtanen M, Elovainio M, Kouvonen A, Väänänen A, Vahtera J (2006). Work stress in the etiology of coronary heart disease – a metaanalysis. Scand J Work Environ Health.

[15] Chang PP, Ford DE, Meoni LA, Wang  N-Y, Klag MJ (2002). Anger in young men and subsequent premature cardiovascular disease: the precursors study. Arch Int Med.

[16] Rezkalla SH, Kloner RA (2007). Cocaine-induced acute myocardial infarction. Clin Med Res.

[17] Mittleman MA, Lewis RA, Maclure M, Sherwood JB, Muller JE (2001). Triggering myocardial infarction by marijuana. Circulation.

[18] Jonsson H, Nived O, Sturfelt G (1989). Outcome in systemic lupus erythematosus: a prospective study of patients from a defined population. Medicine.

[19] Manzi S, Meilahn EN, Rairie JE (1997). Age-specific incidence rates of myocardial infarction and angina in women with systemic lupus erythematosus: comparison with the Framingham Study. Am J Epidemiol.

[20] Currier JS, Taylor A, Boyd F (2003). Coronary heart disease in HIV-infected individuals. J Acquir Immune Defic Syndr.

[21] Triant VA, Lee H, Hadigan C, Grinspoon SK (2007). Increased acute myocardial infarction rates and cardiovascular risk factors among patients with human immunodeficiency virus disease. J Clin Endocrinol Metab.

[22] Hoit BD, Gilpin E, Henning H (1986). Myocardial infarction in young patients: an analysis by age subsets. Circulation.

[23] AlKoubaisyl OK, Mehdi RS, Arem FD, Ahmed IT (1990). Cine angiographic findings in young Iraqi men with first acute myocardial infarction. Cath Cardiovasc Diag.

[24] Yusuf S, Hawken S, Ôunpuu S (2004). Effect of potentially modifiable risk factors associated with myocardial infarction in 52 countries (the INTERHEART study): case–control study. Lancet.

[25] Daviglus ML, Stamler J, Pirzada A (2004). Favorable cardiovascular risk profile in young women and long-term risk of cardiovascular and allcause mortality. J Am Med Assoc.

[26] Bonow RO, Mann DL, Zipes DP, Libby P (2012). Braunwald’s Heart Disease: A Textbook of Cardiovascular Medicine (9th edn). Saunders.

[27] International Diabetes Federation. The IDF consensus worldwide definition of metabolic syndrome, 2006. http://www.idf.org.

[28] Reardon MF, Nestel PJ, Craig IH, Harper RW (1985). Lipoproteins predictors of the severity of coronary artery disease in men and women. Circulation.

[29] Tuzcu EM, Kapadia SR, Tutar E (2001). High prevalence of coronary atherosclerosis in asymptomatic teenagers and young adults evidence from intravascular ultrasound. Circulation.

[30] Patel J, Vyas A, Cruickshank J (2006). Impact of migration on coronary heart disease risk factors: comparison of Gujaratis in Britain and their contemporaries in villages of origin in India. Atherosclerosis.

[31] Vedanthan R, Fuster V (2008). Cardiovascular disease in Sub-Saharan Africa: a complex picture demanding a multifaceted response. Nat Clin Pract Cardiovasc Med.

[32] Vorster HH, Kruger A, Venter CS, Margetts BM, Macintyre UE (2007). Cardiovascular disease risk factors and socio-economic position of Africans in transition: the THUSA study. Cardiovasc J Afr.

[33] Amin AP, Nathan S, Evans AT (2009). The effect of ethnicity on the relationship between premature coronary artery disease and traditional cardiac risk factors among uninsured young adults. Prev Cardiol.

[34] Enas EA (1998). High rates of CAD in Asian Indians in the United States despite intense modification of lifestyle: what next. Curr Sci.

[35] Enas EA, Senthilkumar A (2005). Coronary artery disease in Asian Indians: an update and review. Coron Artery Dis.

[36] Doughty M, Mehta R, Bruckman D (2002). Acute myocardial infarction in the young – The University of Michigan experience. Am Heart J.

[37] Fournier J, Sanchez A, Quero J, Fernandez CJ, González B (1996). Myocardial infarction in men aged 40 years or less: A prospective clinical angiographic study. Clin Cardiol.

[38] Chen L, Chester M, Kaski JC (1995). Clinical factors and angiographic features associated with premature coronary artery disease. Chest.

[39] Klein LW, Agarwal JB, Herlich MB (1987). Prognosis of symptomatic coronary artery disease in young adults aged 40 years or less. Am J Cardiol.

[40] Christus T, Shukkur A, Rashdan I (2011). Coronary artery disease in patients aged 35 or less – a different beast?. Heart Views.

[41] Rissam H, Kishore S, Trehan N (2001). Coronary artery disease in young Indians – the missing link. J Indian Acad Clin Med.

[42] Holbrook J, Grundy S, Hennekens C, Kannel W, Strong J (1984). Cigarette smoking and cardiovascular diseases. A statement for health professionals by a task force appointed by the steering committee of the American Heart Association. Circulation.

[43] Kannel WB, D’Agostino RB, Belanger AJ (1987). Fibrinogen, cigarette smoking, and risk of cardiovascular disease: insights from the Framingham Study. Am Heart J.

[44] McGill HC, McMahan CA, Herderick EE (2002). Obesity accelerates the progression of coronary atherosclerosis in young men. Circulation.

[45] Chua SK, Hung HF, Shyu KG (2010). Acute ST-elevation myocardial infarction in young patients: 15 years of experience in a single center. Clin Cardiol.

[46] Larsen GK, Seth M, Gurm HS (2013). The ongoing importance of smoking as a powerful risk factor for ST-segment elevation myocardial infarction in young patients. J Am Med Assoc.

[47] Ranjith N, Pegoraro R, Rom L, Rajput M, Naidoo D (2003). Lp (a) and apoE polymorphisms in young South African Indians with myocardial infarction. Cardiovasc J S Afr.

[48] Tillin T, Dhutia H, Chambers J (2008). South Asian men have different patterns of coronary artery disease when compared with European men. Int J Cardiol.

[49] Seedat YK, Mayet FGH, Khan S, Somers SR, Joubert G (1990). Risk factors for coronary heart disease in the Indians of Durban. S Afr Med J.

[50] Bachmann JM, Willis BL, Ayers CR, Khera A, Berry JD (2012). Association between family history and coronary heart disease death across long-term follow-up in men: the Cooper Center Longitudinal Study. Circulation.

[51] Chow CK, Islam S, Bautista L (2011). Parental history and myocardial infarction risk across the world: the INTERHEART Study. J Am Coll Cardiol.

[52] Otaki Y, Gransar H, Berman DS (2013). Impact of family history of coronary artery disease in young individuals (from the CONFIRM registry). Am J Cardiol.

[53] Bao W, Srinivasan SR, Wattigney WA, Berenson GS (1995). The relation of parental cardiovascular disease to risk factors in children and young adults: The Bogalusa Heart Study. Circulation.

[54] Ford ES, Giles WH, Dietz WH (2002). Prevalence of the metabolic syndrome among US adults: findings from the third National Health and Nutrition Examination Survey. J Am Med Assoc.

[55] Azizi F, Salehi P, Etemadis A (2003). Prevalence of metabolic syndrome in an urban population: Tehran Lipid and Glucose Study. Diabetes Res Clin Pract.

[56] Ranjith N, Pegoraro RJ, Naidoo DP, Esterhuizen TM (2007). Metabolic syndrome in young Asian Indians with myocardial infarction. Cardiovasc J Afr.

[57] Wadhwa A, Avasthi R, Ghambhir J, Dwivedi S (2013). To study the prevalence and profile of metabolic syndrome, levels of hs-CRP, LP(a) and serum ferritin in young Indian patients (≤ 45 years) with acute myocardial infarction. J Assoc Phys India.

[58] Stamler J, Stamler R, Neaton JD (1999). Low risk-factor profile and long-term cardiovascular and noncardiovascular mortality and life expectancy: findings for 5 large cohorts of young adult and middle-aged men and women. J Am Med Assoc.

[59] Wolfe M, Vacek J (1988). Myocardial infarction in the young. Angiographic features and risk factor analysis of patients with myocardial infarction at or before the age of 35 years. Chest.

